# A minimal growth medium for the basidiomycete *Pleurotus sapidus* for metabolic flux analysis

**DOI:** 10.1186/s40694-014-0009-4

**Published:** 2014-12-05

**Authors:** Marco A Fraatz, Stefanie Naeve, Vanessa Hausherr, Holger Zorn, Lars M Blank

**Affiliations:** 1 Institute of Food Chemistry and Food Biotechnology, Justus Liebig University Giessen, Heinrich-Buff-Ring 58, Giessen, 35392 Germany; 2 Laboratory of Technical Biochemistry, TU Dortmund, Dortmund, 44221 Germany; 3 IfADo - Leibniz Research Center for Working Environment and Human Factors, Ardeystr. 67, Dortmund, 44139 Germany; 4 iAMB - Institute of Applied Microbiology, ABBt - Aachen Biology and Biotechnology, RWTH Aachen University, Worringer Weg 1, Aachen, 52074 Germany

**Keywords:** Basidiomycete, Metabolic flux analysis, Minimal growth medium, Central carbon metabolism, Submerged culture, 13C-flux analysis

## Abstract

**Background:**

*Pleurotus sapidus* secretes a huge enzymatic repertoire including hydrolytic and oxidative enzymes and is an example for higher basidiomycetes being interesting for biotechnology. The complex growth media used for submerged cultivation limit basic physiological analyses of this group of organisms. Using undefined growth media, only little insights into the operation of central carbon metabolism and biomass formation, *i.e.*, the interplay of catabolic and anabolic pathways, can be gained.

**Results:**

The development of a chemically defined growth medium allowed rapid growth of *P. sapidus* in submerged cultures. As *P. sapidus* grew extremely slow in salt medium, the co-utilization of amino acids using ^13^C-labelled glucose was investigated by gas chromatography–mass spectrometry (GC-MS) analysis. While some amino acids were synthesized up to 90% *in vivo* from glucose (*e.g.,* alanine), asparagine and/or aspartate were predominantly taken up from the medium. With this information in hand, a defined yeast free salt medium containing aspartate and ammonium nitrate as a nitrogen source was developed. The observed growth rates of *P. sapidus* were well comparable with those previously published for complex media. Importantly, fast growth could be observed for 4 days at least, up to cell wet weights (CWW) of 400 g L^-1^. The chemically defined medium was used to carry out a ^13^C-based metabolic flux analysis, and the *in vivo* reactions rates in the central carbon metabolism of *P. sapidus* were investigated. The results revealed a highly respiratory metabolism with high fluxes through the pentose phosphate pathway and TCA cycle.

**Conclusions:**

The presented chemically defined growth medium enables researchers to study the metabolism of *P. sapidus*, significantly enlarging the analytical capabilities. Detailed studies on the production of extracellular enzymes and of secondary metabolites of *P. sapidus* may be designed based on the reported data.

**Electronic supplementary material:**

The online version of this article (doi:10.1186/s40694-014-0009-4) contains supplementary material, which is available to authorized users.

## Background

Higher basidiomycetes contribute to the human diet in many societies and are increasingly investigated for their potential in biotechnology. The latter is mainly motivated by the huge hydrolytic potential of this large group of organisms, of which many are saprophytic. Application examples of fungal enzymes include the degradation of biomass [1], the production of fine chemicals including *e.g.* norisoprenoids [2], monoterpenes [3],[4], and cyathane type diterpenoids [5]. While the enzymatic repertoire of some higher basidiomycetes has been investigated in detail [6], the nutritional requirements of higher basidiomycetes are often not. The mycelium of filamentous fungi, like *Pleurotus sapidus*, can be grown in submerged cultures utilizing shake flasks or bioreactors. In general, glucose acts as the major carbon source in the growth media of higher basidiomycetes, which usually contain additional complex ingredients, like yeast extract, malt extract, or soya peptone. By-products of the food industry can be added to liquid cultures of basidiomycetes as the only carbon source and to promote the biotechnological production of complex flavor mixtures [7]. Inorganic salts, amino acids, vitamins, and trace element solutions are often added to the media.

These complex media can support biomass formation, with specific growth rates of 0.02 h^−1^ and higher [8]. Indeed, the growth rate is of major importance for experimenters and it is thus optimized to allow rapid and reproducible experiments. However, the complex nature of the growth media usually used makes it difficult to determine substrate uptake rates and hence, the true demand of the mycelium grown in submerged culture is mainly unknown. In the literature, only very few reports are found covering flux analysis of basidiomycetes [9],[10]. None of them covers filamentous species, but the basidiomycetous yeast *Phaffia rhodozyma* has been examined*.* Therefore, a chemically defined medium that allowed high growth rates and hence metabolic studies was developed. With this medium, the intracellular flux distribution in a higher basidiomycete by means of ^13^C-tracer based flux analysis was estimated for the first time. The results revealed a highly respiratory metabolism, with significant contribution of glucose catabolism via the pentose phosphate pathway. The analytical possibilities reported here open new potentials for higher basidiomycete bio(techno)logy.

## Results and discussion

### Development of a minimal medium for the submerged cultivation of *P. sapidus*

**Figure 1:**
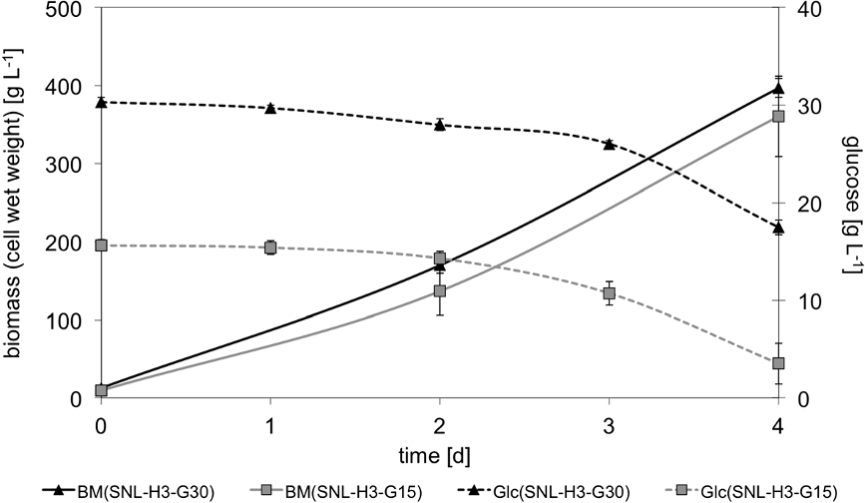
**Growth kinetics of** ***P. sapidus*** **in complex standard medium.** Initial glucose concentration of standard medium 30 g L^-1^ (SNL-H3-G30) and 15 g L^-1^ (SNL-H3-G15), respectively, BM: biomass, Glc: glucose, *cf.* Table 1 for detailed medium composition.

**Figure 2:**
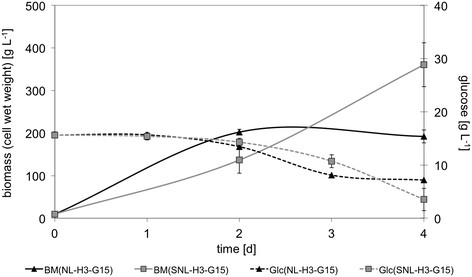
**Growth kinetics of** ***P. sapidus*** **in dependence on Asn supplementation.** BM: biomass, Glc: glucose, SNL-H3-G15: standard medium, NL-H3-G15: without Asn, *cf.* Table 1 for detailed medium composition.

**Figure 3:**
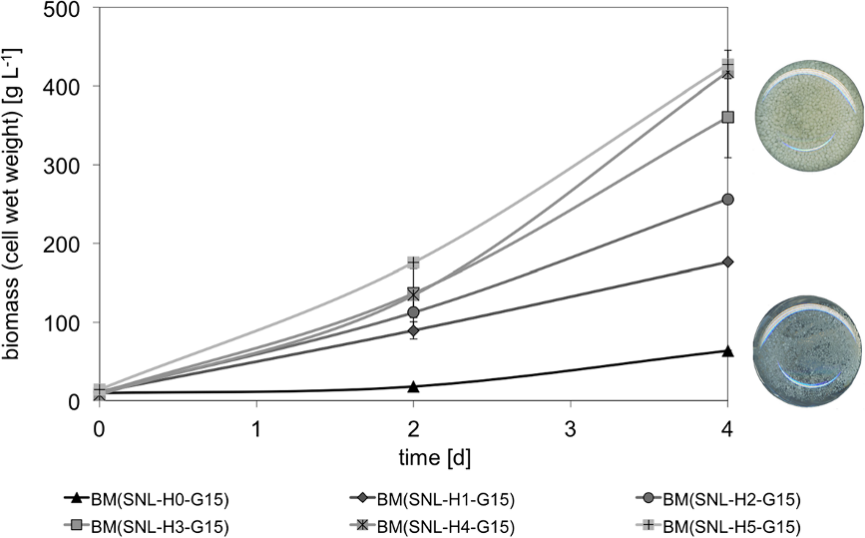
**Growth of** ***P. sapidus*** **in dependence on yeast extract supplementation.** Left: Growth of *P. sapidus* in dependence on yeast extract supplementation; H0 - H5 equates to 0–5 g L^-1^ yeast extract, *cf.* Table 1 for detailed medium composition. Right: Visual comparison of *P. sapidus* grown for 4 days in standard nutrition medium with 3 g L^-1^ (SNL-H3-G15, top) and 0 g L^-1^ (SNL-H0-G15, bottom) yeast extract.

Basidiomycetes like *P. sapidus* are typically grown submerged in complex culture media. To investigate growth kinetics and cellular physiology in detail, minimal media are the first choice in many areas of microbiology. For higher fungi like *P. sapidus* minimal media were not readily available. Hence, a defined minimal medium was developed starting from a commonly used complex medium called standard nutrition solution (SNL-H3-G30, *cf.* Table 1). SNL-H3-G30 is derived from Sprecher’s medium [11] by addition of yeast extract. Like many other basidiomycetes, *P. sapidus* grows only poorly in unmodified Specher’s medium but very well in the modified one. To benchmark growth, *P. sapidus* was therefore cultivated in SNL-H3-G30. During the first four days of growth *P. sapidus* consumed approximately 15 g L^-1^ glucose. Reducing the sugar concentration of the culture medium by a factor of two did not influence the biomass production significantly (Figure 1). After replacing the standard nutrition solution’s (SNL-H3-G15) nitrogen source asparagine by ammonium nitrate (NL-H3-G15, Table 1) the growth rate was initially higher compared to SNL-H3-G15, but stalled after 48 h (Figure 2). To investigate if the availability of nitrogen caused reduced growth, different ammonium nitrate concentrations (1.2 - 7.2 g L^-1^) were evaluated. The production of biomass of *P. sapidus* was not effected (data not shown). In contrast, the concentration of yeast extract in the culture medium correlated directly with the biomass production of *P. sapidus* (Figure 3). Without the addition of yeast extract (SNL-H0-G15) very limited growth was observed in standard nutrition solution (Figure 3), as well as in medium with ammonium nitrate as the nitrogen source (NL-H0-G15) (Table 2).

**Table 1:** **Composition of culture media**

	Glc [g L ^-1^ ]	Asn [g L ^-1^ ]	Asp [g L ^-1^ ]	NH _4_ NO _3_ [g L ^-1^ ]	KH _2_ PO _4_ [g L ^-1^ ]	MgSO _4_ [g L ^-1^ ]	yeast [g L ^-1^ ]	TE [mL L ^-1^ ]	vit [mL L ^-1^ ]
SNL-H3-G30	30.0	4.5	0.0	0.0	1.5	1.0	3.0	1.0	0.0
SNL-H5-G15	15.0	4.5	0.0	0.0	1.5	1.0	5.0	1.0	0.0
SNL-H4-G15	15.0	4.5	0.0	0.0	1.5	1.0	4.0	1.0	0.0
SNL-H3-G15	15.0	4.5	0.0	0.0	1.5	1.0	3.0	1.0	0.0
SNL-H2-G15	15.0	4.5	0.0	0.0	1.5	1.0	2.0	1.0	0.0
SNL-H1-G15	15.0	4.5	0.0	0.0	1.5	1.0	1.0	1.0	0.0
SNL-H0-G15	15.0	4.5	0.0	0.0	1.5	1.0	0.0	1.0	0.0
NL-H3-G15	15.0	0.0	0.0	2.4	1.5	1.0	3.0	1.0	0.0
NL-H2-G15	15.0	0.0	0.0	2.4	1.5	1.0	2.0	1.0	0.0
NL-H1-G15	15.0	0.0	0.0	2.4	1.5	1.0	1.0	1.0	0.0
NL-H0-G15	15.0	0.0	0.0	2.4	1.5	1.0	0.0	1.0	0.0
NL-D5-G30	15.0	0.0	4.8	2.4	1.5	1.0	0.0	1.0	10.0 BME
**NL-D5-G15**	15.0	0.0	4.8	2.4	1.5	1.0	0.0	1.0	10.0 BME
NL-D0.4-G15	15.0	0.0	0.4	2.4	1.5	1.0	0.0	1.0	1.0 VER

The pH was adjusted to 6.0 with 1 M NaOH prior to sterilization; Glc: D-glucose monohydrate, Asn: L-asparagine, Asp: L-aspartic acid, MgSO_4_: magnesium sulfate heptahydrate, yeast: yeast extract, TE: trace elements solution (5 mg L^-1^ CuSO_4_·5 H_2_O, 80 mg L^-1^ FeCl_3_·6 H_2_O, 90 mg L^-1^ ZnSO_4_·7 H_2_O, 30 mg L^-1^ MnSO_4_·H_2_O, and 0.4 g L^-1^ EDTA), vit: BME vitamins solution or after [12] (VER), medium NL-D5-G15 (highlighted in bold) was used for flux analysis (*cf*. Figure 6).

**Table 2:** **Biomass (cell wet weight) of** ***P. sapidus*** **after 4 days when grown in culture media with ammonium nitrate as nitrogen source and different yeast extract concentrations**

Medium name	Yeast extract [g L ^-1^ ]	Cell wet weight [g L ^-1^ ]
NL-H0-G15	0.0	31
NL-H1-G15	1.0	83
NL-H2-G15	2.0	157
NL-H3-G15	3.0	192

*Cf.* Table 1 for detailed medium composition.

In addition, the influence of thiamine, a vitamin mixture (after [12]), as well as of different trace element solutions (after [11] and [12], respectively) on the growth rate was investigated. No significant effects on the rate of growth or the final biomass concentrations were observed (data not shown).

**Figure 4:**
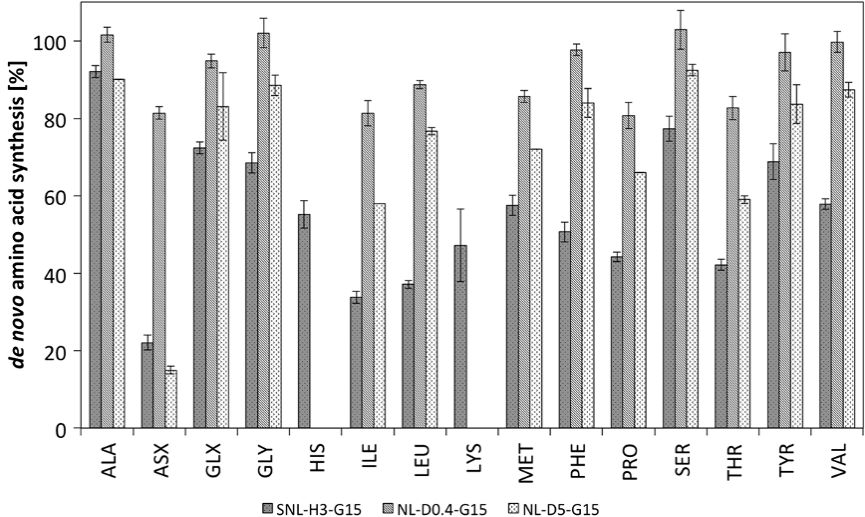
***De novo*** **synthesis of amino acids in** ***P. sapidus*** **.** The bars represent the relative amount of *de novo* synthesized amino acids during growth in standard nutrition solution (SNL-H3-G15) and two chemical defined media with different aspartate concentrations (0.4 g L-1: NL-D0.4-G15; 4.8 g L-1: NL-D5-G15). The contribution of *de novo* synthesis was estimated from the amount of label measured in the amino acids, which originated from 50% [U-^13^C]-labeled glucose as main carbon source. Error bars represent the range of duplicates.

To determine which amino acids are used by *P. sapidus* as co-substrates and to which extent, the basidiomycete was grown in yeast containing standard nutrition solution (SNL-H3-G15) with a mixture of [U-^13^C]-glucose and unlabeled glucose (50:50, w/w) as its carbon source. After harvesting the fungus, hydrolysis, and derivatization, the fractional labeling of the amino acids (Ala, Asx, Glx, Gly, His, Ile, Leu, Lys, Met, Phe, Pro, Ser, Thr, Tyr, and Val) was determined by GC-MS. The *de novo* synthesis of amino acids from glucose was between 22% (Asx) and 92% (Ala) (Figure 4). Thus, all amino acids were metabolized by *P. sapidus*, however to very different proportions.

To further simplify the medium, single amino acids as well as selected combinations of amino acids were tested for their growth rate promotion in yeast free media containing ammonium nitrate as an additional nitrogen source. All combinations without aspartate resulted in poor growth rates and biomass concentrations (data not shown). Therefore, medium NL-D5-G15 containing aspartate (4.8 g L^-1^), salts (NH_4_NO_3_, KH_2_PO_4_, and MgSO_4_), vitamins, trace elements, and 15 g L^-1^ glucose was selected as the simplest chemically defined minimal medium for further investigations.

**Figure 5:**
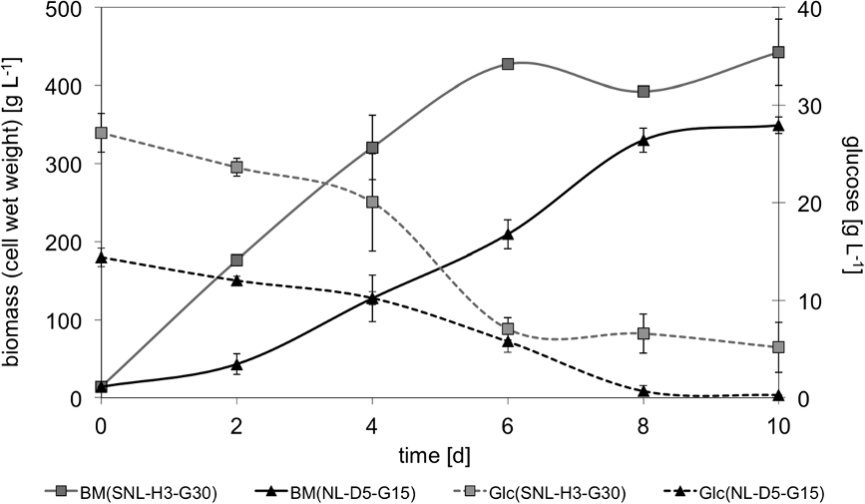
**Growth kinetics of** ***P. sapidus*** **in developed minimal medium (NL-D5-G15) in comparison to standard nutrition solution (SNL-H3-G30).** BM: biomass, Glc: glucose, *cf.* Table 1 for detailed medium composition.

Under all conditions tested, *P. sapidus* grew filamentous. The mycelium rapidly formed pellets, which increased over time in size (*cf.* Figure 3). Strategies to avoid pellet formation are discussed in the literature [13],[14] and might be applied to the newly developed growth medium in future. The resulting salt medium with the single amino acid aspartate allowed for a consistent and rapid growth for 6 to 8 days (Figure 5), and could therefore be used for quantitative physiological experiments.

### Use of the minimal medium for quantitative physiology of *P. sapidus*

**Figure 6:**
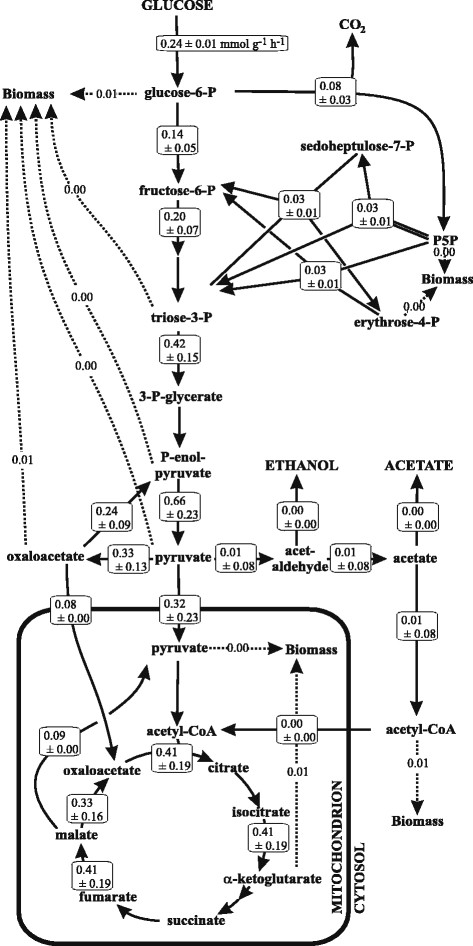
**Absolute metabolic fluxes of** ***P. sapidus.*** *P. sapidus* was grown in medium NL-D5-G15, *cf.* Table 1 for detailed medium composition.

For the sole addition of aspartate, the *de novo* synthesis of amino acids was quantified by ^13^C-labeling of glucose (Figure 4) at two different aspartate concentrations (NL-D0.4-G15, NL-D5-G15, Table 1). The addition of only 0.4 g L^-1^ aspartate resulted in minor use of this amino acid as additional carbon source. Only Asx, Ile, Pro, and Thr were partially (about 20%) synthesized from aspartate, while glucose was the main source of the respective carbon skeleton. Indeed, aspartate is the precursor for threonine and isoleucine synthesis. The absence of unlabeled carbon in glycine suggests that a threonine aldolase, catalyzing the synthesis of glycine from threonine while producing acetaldehyde is not present or not active in *P. sapidus* under the investigated conditions. The amino acids derived from ketoglutarate (Glx, Pro) were partially synthesized from aspartate. Aspartate is readily deaminated to oxaloacetate explaining the contribution to TCA cycle intermediates. No unlabeled carbon was observed in pyruvate derived amino acids (*e.g.*, Ala, Val), indicating that gluconeogenic reactions are absent during growth on glucose. The contribution of 10% of aspartate to the mainly pyruvate derived amino acid leucine is not readily explained, as pyruvate is fully labeled (*e.g.*, Ala). In addition, two carbon atoms of leucine originate from acetyl-CoA. Acetyl-CoA can either originate from cytosolic or mitochondrial pyruvate. The latter is synthesized via the pyruvate dehydrogenase complex, although contributions from the malic enzyme (malate to pyruvate) were reported for ascomycetous [10],[15],[16], but not basidiomycetous yeasts. Indeed, when performing a ^13^C-based metabolic flux analysis, a malic enzyme activity was observed in *P. sapidus* (Figure 6). In general, with increased aspartate concentrations (4.8 g L^-1^), the contribution to amino acid *de novo* synthesis increased slightly. The exception was the synthesis of Asx, which originated to more than 80% from aspartate taken up from the medium, and less distinct the synthesis of isoleucine and threonine (about 40%).

Performing a ^13^C-flux analysis experiment with the defined medium NL-D5-G15 (Table 1) allowed for the quantification of the glucose uptake rate and specific growth rate with 0.24 mmol g^-1^ h^-1^ and 0.048 h^-1^, respectively. These values are low when compared to previous reports on ascomycetes. Glucose was catabolized via glycolysis and up to 35% via the pentose phosphate pathway. In the basidiomycetous yeast *Phaffia rhodozyma,* glucose was catabolized via the pentose phosphate pathway up to 65% [10]. No by-products like ethanol, acetate or glycerol were observed in *P. sapidus* cultures (data not shown). These result in combination with a highly active TCA cycle (more than 80% of the oxaloacetate originated from the TCA cycle, while less than 20% originated from the anaplerotic reaction catalyzed by the pyruvate carboxylase) strongly indicated that the metabolism of *P. sapidus* is fully respiratory under the growth conditions tested here.

The absolute fluxes indicated a considerable flux to biomass. This is also in agreement with the flux through the pentose phosphate pathway as not only biomass precursors like ribose and erythrose-4P for nucleic and amino acids synthesis, respectively, but also the anabolic demand for NADPH can be met via the oxidative branch of this pathway. Indeed, the flux through the pentose phosphate pathway was previously linked to the biomass yield in ascomycetes [16].

## Conclusions

The presented results allow for experiments with *P. sapidus* growing submerged in a chemically defined medium. This enables researchers to study the biology of *P. sapidus* (and possibly other mushrooms) in the context of metabolism, significantly enlarging the analytical capabilities. While the information of the respiratory capabilities is highly interesting, the low overall metabolic activity most likely requires modifications of *P. sapidus* as a production host in industrial biotechnology. With this information in hand, *e.g.*, detailed induction studies of hydrolytic enzymes of *P. sapidus* can be designed.

## Methods

### Chemicals

Copper(II) sulfate pentahydrate, iron(III) chloride hexahydrate, and zinc sulfate heptahydrate were purchased from AppliChem (Darmstadt, Germany), D-glucose [U-^13^C] from EURISO-TOP (Gif-sur-Yvette, France); D-glucose monohydrate and L-aspartic acid were obtained from Carl Roth GmbH (Karlsruhe, Germany), EDTA from Fluka (Buchs, Germany); agar, L-asparagine, magnesium sulfate heptahydrate, manganese(II) sulfate monohydrate, and yeast extract were purchased from Serva (Heidelberg, Germany); Basal Medium Eagle (BME) vitamins solution was purchased from Sigma (Steinheim, Germany).

### Microorganism

The filamentous fungus *Pleurotus sapidus* was obtained from the German Collection of Microorganisms and Cell Cultures (DSMZ 8266), Brunswick, Germany.

### Cultivation of *P. sapidus*

Stock cultures were maintained on agar plates containing standard nutrition solution (SNL-H3-G30, Table 1) and 15 g L^-1^ agar. The stock cultures were stored at 4°C until usage.

Precultures were grown aerobically in standard nutrition solution (SNL-H3-G30, Table 1) after transferring 1 cm^2^ agar plugs from the leading mycelial edge of the stock cultures followed by homogenization using an Ultra Turrax homogenizer (IKA, Staufen, Germany). The submerged cultures (200 mL medium) were kept on a rotary shaker (25 mm shaking diameter; Multitron, Infors, Einsbach, Germany) at 150 rpm and 24°C in Erlenmeyer flasks (500 mL) for 4 days in darkness. The precultures were centrifuged for 10 min (3375 *× g*, 4°C), and the supernatant was decanted. The remaining pellets were resuspended in the same volume of distilled water and centrifuged again for 10 min (3375 *× g*, 4°C). This procedure was repeated twice. Subsequently to the last centrifugation step the pellets were dispersed in the main culture medium (Table 1) and homogenized using an Ultra Turrax homogenizer. For the main cultures 40 mL medium was inoculated with 4 mL homogenized preculture broth in Erlenmeyer flasks (100 mL) and incubated on a rotary shaker (25 mm shaking diameter, 150 rpm, 24°C) for 4 to 10 days.

### ^13^C-based carbon flux analysis

The GC-MS data represent sets of ion clusters, each showing the distribution of mass isotopomers of a given amino acid fragment. For each fragment *α*, one mass isotopomer distribution vector (MDV) was assigned,

1 $$MDV_{a}=\left[\begin{array}{l} \left(m_{0}\right)\\ \left(m_{1}\right)\\ \left(m_{2}\right)\\ \ldots \\ \left(m_{n}\right) \end{array}\right]\mathrm{with}\sum m_{i}=1$$

with m0 being the fractional abundance of the lowest mass and m_i_ > 0 the abundances of molecules with higher masses. To obtain the exclusive mass isotope distribution of the carbon skeleton, corrections for naturally occurring isotopes in the derivatization reagent and the amino acids were performed as described previously [17],[18], followed by the calculations of the mass distribution vectors of the amino acids (MDV_AA_) and the metabolites (MDV_M_). Metabolic flux ratios were calculated from the MDV_M_ as described in detail by Nanchen *et al.* [19] using Fiat Flux [20]. Absolute values of intracellular fluxes were calculated with a flux model from the yeast *Saccharomyces cerevisiae* that comprised all major pathways of central carbon metabolism [21]. The error minimization was carried out as described by Fischer *et al.* [18].

### Analytical methods

#### Determination of cell wet weight

The culture broth was centrifuged for 10 min (3375 *×* *g*, 4°C), and the supernatant was replaced by the same volume of distilled water. The mycelium was resuspended and centrifuged. This washing step was repeated twice. Afterwards, the supernatant was discarded, and the weight of the remaining mycelium was determined.

#### Determination of glucose

The D-glucose concentration in the culture supernatant was determined enzymatically by means of an enzymatic D-glucose assay (R-Biopharm AG, Darmstadt, Germany) according to manufacturer’s instruction.

#### Determination of the ^13^C-labeling patterns of the proteinogenic amino acids

The glucose used in shake flasks experiments was a mixture of 50% (n/n) uniformly labeled [U-^13^C]-glucose and 50% (n/n) naturally labeled glucose. The biomass was washed twice with 0.9% NaCl and hydrolyzed with 150 μL of 6 M HCl for 15–24 h at 105°C. The hydrolyzate was dried by heating the vial to 85°C under a constant flow of air. The hydrolyzate was dissolved in 50 μL dimethyl formamide and transferred to a new vial. The amino acids were silylated by addition of 50 μL N-methyl-N(*tert*-butyldimethylsilyl)-trifluoroacetamide and subsequently incubated at 85°C for 60 min. One μL of this mixture was injected into a Varian GC 3800 gas chromatograph, equipped with a Varian MS/MS 1200 triple quadrupole mass spectrometer (Varian Deutschland, Darmstadt, Germany). The derivatized amino acids were separated on a FactorFour VF-5ms column (30 m × 0.25 mm ID, 0.25 μm film thickness; Varian Deutschland) at a constant flow rate of 1 mL helium (5.0) min^-1^. The split ratio was 1:25 and the inlet temperature was set to 250°C. The temperature of the GC oven was kept constant for 2 min at 150°C and afterwards increased to 250°C with a gradient of 3°C min^-1^. The temperatures of the transfer line and the source were 280°C and 250°C, respectively. Ionization was performed by electron impact ionization at -70 eV. For enhanced detection, a selected ion monitoring time segment was defined for every amino acid [22]. GC-MS raw data were analyzed using the Workstation MS Data Review (Varian Deutschland).

#### Determination of *de novo* amino acid synthesis

Intracellular *de novo* amino acid synthesis was determined for the amino acids alanine, aspartate, glutamate, glycine, histidine, isoleucine, leucine, lysine, methionine, phenylalanine, proline, serine, threonine, tyrosine, and valine as previously reported in [23]. The percentages of *de novo* synthesized amino acids correspond to the ^13^C-labeling in the amino acids derived from ^13^C labeled glucose. The unlabeled fraction corresponds to the amount of unlabeled amino acid, which was taken up from the medium. GC-MS analysis based on proteinogenic amino acids is able to detect 15 of the 20 proteinogenic amino acids. Arginine was omitted because rearrangements during electron impact ionization obscure its fragmentation pattern. Cysteine and tryptophan are oxidatively destroyed during acid hydrolysis, and asparagine and glutamine are deamidated to aspartate and glutamate, respectively [24]. The mixtures of asparagine/aspartate and glutamine/glutamate were subsequently referred to as ASX and GLX, respectively. Labeling patterns were analyzed using the software FiatFlux [20].

## Authors’ original submitted files for images

Below are the links to the authors’ original submitted files for images. Authors’ original file for figure 1
Authors’ original file for figure 2
Authors’ original file for figure 3
Authors’ original file for figure 4
Authors’ original file for figure 5
Authors’ original file for figure 6

## Abbreviations

Ala: Alanine
Asx: Asparagine/aspartate
Asn: Asparagine
Asp: Aspartic acid
BME: Basal medium eagle
CWW: Cell wet weight
EDTA: Ethylenediaminetetraacetic acid
GC: Gas chromatography
GC-MS: Gas chromatography–mass spectrometry
Glc: Glucose
Glx: Glutamine/glutamate
Gly: Glycine
His: Histidine
ID: Inner diameter
Ile: Isoleucine
Leu: Leucine
Lys: Lysine
MDV: Mass isotopomer distribution vector
Met: Methionine
Phe: Phenylalanine
Pro: Proline
Ser: Serine
SNL: Standard nutrition solution
TCA: Tricarboxylic acid cycle
Thr: Threonine
TE: Trace elements solution
Tyr: Tyrosin
Val: Valine
vit: Vitamins solution

## References

[1] Ichinose H (2013). Cytochrome P450 of wood-rotting basidiomycetes and biotechnological applications. Biotechnol Appl Biochem.

[2] Zelena K, Hardebusch B, Hülsdau B, Berger RG, Zorn H (2009). Generation of norisoprenoid flavors from carotenoids by fungal peroxidases. J Agric Food Chem.

[3] Krings U, Lehnert N, Fraatz MA, Hardebusch B, Zorn H, Berger RG (2009). Autoxidation versus biotransformation of *α*-pinene to flavors with *Pleurotus sapidus*: regioselective hydroperoxidation of *α*-pinene and stereoselective dehydrogenation of verbenol. J Agric Food Chem.

[4] Fraatz MA, Riemer SJL, Stöber R, Kaspera R, Nimtz M, Berger RG, Zorn H (2009). A novel oxygenase from *Pleurotus sapidus* transforms valencene to nootkatone. J Mol Catal B Enzym.

[5] Bhandari DR, Shen T, Römpp A, Zorn H, Spengler B (2014). Analysis of cyathane-type diterpenoids from *Cyathus striatus* and *Hericium erinaceus* by high-resolution MALDI MS imaging. Anal Bioanal Chem.

[6] Bouws H, Wattenberg A, Zorn H (2008). Fungal secretomes—nature’s toolbox for white biotechnology. Appl Microbiol Biotechnol.

[7] Bosse AK, Fraatz MA, Zorn H (2013). Formation of complex natural flavors by biotransformation of apple pomace with basidiomycetes. Food Chem.

[8] Tlecuitl-Beristain S, Sánchez C, Loera O, Robson GD, Díaz-Godínez G (2008). Laccases of *Pleurotus ostreatus* observed at different phases of its growth in submerged fermentation: production of a novel laccase isoform. Microbiol Res.

[9] Dong Q-L, Zhao X-M, Ma H-W, Xing X-Y, Sun N-X (2006). Metabolic flux analysis of the two astaxanthin-producing microorganisms *Haematococcus pluvialis* and *Phaffia rhodozyma* in the pure and mixed cultures. Biotechnol J.

[10] Cannizzaro C, Christensen B, Nielsen J, von Stockar U (2004). Metabolic network analysis on *Phaffia rhodozyma* yeast using ^13^C–labeled glucose and gas chromatography–mass spectrometry. Metab Eng.

[11] Sprecher E (1959). Über die Guttation bei Pilzen. Planta.

[12] Verduyn C, Postma E, Scheffers WA, Van Dijken JP (1992). Effect of benzoic acid on metabolic fluxes in yeasts: a continuous-culture study on the regulation of respiration and alcoholic fermentation. Yeast.

[13] Kaup BA, Ehrich K, Pescheck M, Schrader J (2008). Microparticle-enhanced cultivation of filamentous microorganisms: increased chloroperoxidase formation by *Caldariomyces fumago* as an example. Biotechnol Bioeng.

[14] Walisko R, Krull R, Schrader J, Wittmann C (2012). Microparticle based morphology engineering of filamentous microorganisms for industrial bio-production. Biotechnol Lett.

[15] Blank LM, Sauer U (2004). TCA cycle activity in *Saccharomyces cerevisiae* is a function of the environmentally determined specific growth and glucose uptake rates. Microbiology.

[16] Blank LM, Lehmbeck F, Sauer U (2005). Metabolic-flux and network analysis in fourteen hemiascomycetous yeasts. FEMS Yeast Res.

[17] Fischer E, Sauer U (2003). Metabolic flux profiling of *Escherichia coli* mutants in central carbon metabolism using GC-MS. Eur J Biochem.

[18] Fischer E, Zamboni N, Sauer U (2004). High-throughput metabolic flux analysis based on gas chromatography–mass spectrometry derived ^13^C constraints. Anal Biochem.

[19] Nanchen A, Fuhrer T, Sauer U (2007). Determination of metabolic flux ratios from ^13^C-experiments and gas chromatography–mass spectrometry data. Methods Mol Biol.

[20] Zamboni N, Fischer E, Sauer U (2005). FiatFlux – a software for metabolic flux analysis from ^13^C-glucose experiments. BMC Bioinformatics.

[21] Blank LM, Kuepfer L, Sauer U (2005). Large-scale ^13^C-flux analysis reveals mechanistic principles of metabolic network robustness to null mutations in yeast. Genome Biol.

[22] Wittmann C (2007). Fluxome analysis using GC-MS. Microb Cell Fact.

[23] Heyland J, Fu J, Blank LM, Schmid A (2010). Quantitative physiology of *Pichia pastori* s during glucose-limited high-cell density fed-batch cultivation for recombinant protein production. Biotechnol Bioeng.

[24] Dauner M, Sauer U (2000). GC-MS analysis of amino acids rapidly provides rich information for isotopomer balancing. Biotechnol Progress.

