# Supplementary figures and images for: A minimal growth medium for the basidiomycete Pleurotus sapidus for metabolic flux analysis
Source: Fungal Biol Biotechnol. 2014 Dec 5;1:9. doi: 10.1186/s40694-014-0009-4 (PMC5611629; doi:10.1186/s40694-014-0009-4)

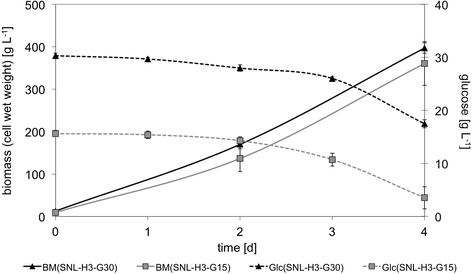

Supplement: Supplementary file 1 — Authors’ original file for figure 1 [file 40694_2014_9_MOESM1_ESM.gif]

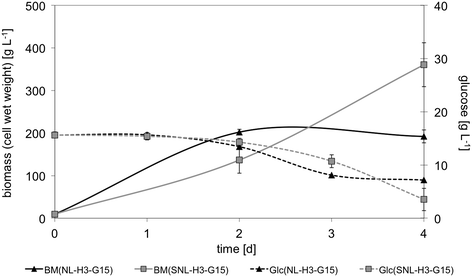

Supplement: Supplementary file 2 — Authors’ original file for figure 2 [file 40694_2014_9_MOESM2_ESM.gif]

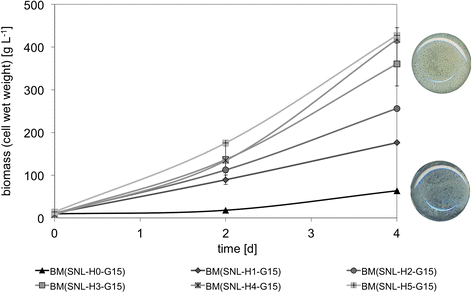

Supplement: Supplementary file 3 — Authors’ original file for figure 3 [file 40694_2014_9_MOESM3_ESM.gif]

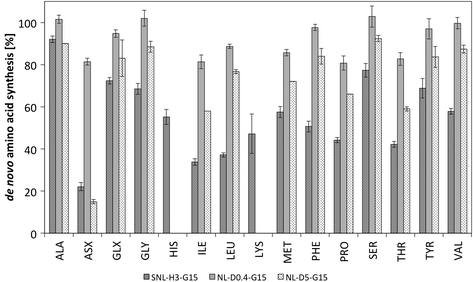

Supplement: Supplementary file 4 — Authors’ original file for figure 4 [file 40694_2014_9_MOESM4_ESM.gif]

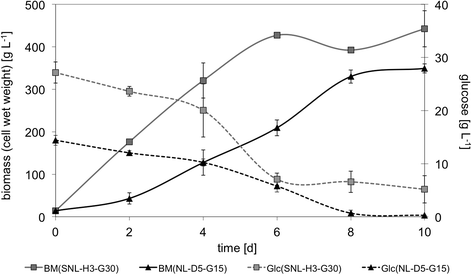

Supplement: Supplementary file 5 — Authors’ original file for figure 5 [file 40694_2014_9_MOESM5_ESM.gif]

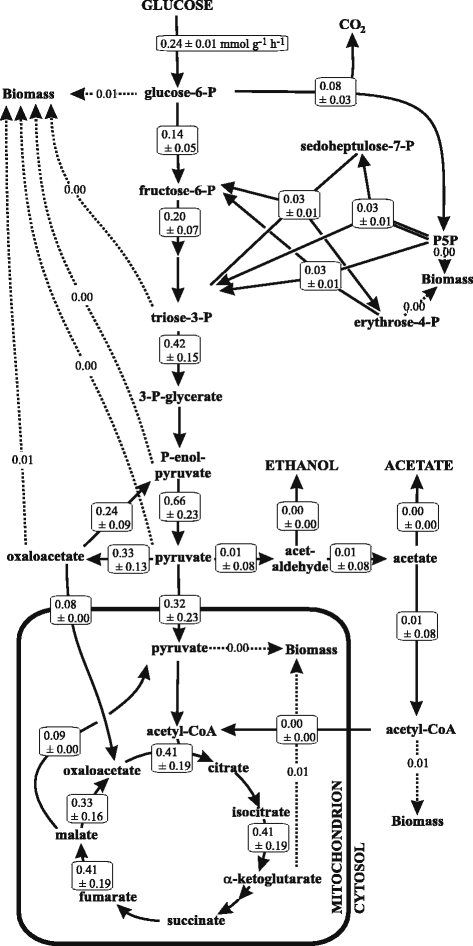

Supplement: Supplementary file 6 — Authors’ original file for figure 6 [file 40694_2014_9_MOESM6_ESM.gif]
